# The accuracy of predicting hospital admission by emergency medical service and emergency department personnel compared to the prehospital MEWS: a prospective multicenter study

**DOI:** 10.1186/s12873-024-01031-9

**Published:** 2024-07-09

**Authors:** Lars I. Veldhuis, Laura van der Weide, Prabath Nanayakkara, Jeroen Ludikhuize

**Affiliations:** 1 Emergency Department, Amsterdam UMC, Location Academic Medical Centre, Amsterdam, the Netherlands; 2 Department of Anesthesiology, Erasmus Medical Center, Rotterdam, the Netherlands; 3 Section General and Acute Internal Medicine, Department of Internal Medicine, Amsterdam Public Health Research Institute, Amsterdam UMC, Amsterdam, the Netherlands; 4 Department of Intensive Care medicine, HagaHospital, the Hague, the Netherlands

**Keywords:** Hospital admission, Deterioration, Emergency department, Early warning score

## Abstract

**Introduction:**

Overcrowding in the emergency department (ED) is a global problem. Early and accurate recognition of a patient’s disposition could limit time spend at the ED and thus improve throughput and quality of care provided. This study aims to compare the accuracy among healthcare providers and the prehospital Modified Early Warning Score (MEWS) in predicting the requirement for hospital admission.

**Methods:**

A prospective, observational, multi-centre study was performed including adult patients brought to the ED by ambulance. Involved Emergency Medical Service (EMS) personnel, ED nurses and physicians were asked to predict the need for hospital admission using a structured questionnaire. Primary endpoint was the comparison between the accuracy of healthcare providers and prehospital MEWS in predicting patients’ need for hospital admission.

**Results:**

In total 798 patients were included of whom 393 (49.2%) were admitted to the hospital. Sensitivity of predicting hospital admission varied from 80.0 to 91.9%, with physicians predicting hospital admission significantly more accurately than EMS and ED nurses (*p* < 0.001). Specificity ranged from 56.4 to 67.0%. All healthcare providers outperformed MEWS ≥ 3 score on predicting hospital admission (sensitivity 80.0-91.9% versus 44.0%; all *p* < 0.001). Predictions for ward admissions specifically were significantly more accurate than MEWS (specificity 94.7–95.9% versus 60.6%, all *p* < 0.001).

**Conclusions:**

Healthcare providers can accurately predict the need for hospital admission, and all providers outperformed the MEWS score.

**Supplementary Information:**

The online version contains supplementary material available at 10.1186/s12873-024-01031-9.

## Introduction

Emergency departments (ED) all around the world face an increasing number of patients, leading to overcrowding and potentially lower quality of care. [1–3] The Netherlands is facing a similar development. [4] Both emergency medical service (EMS) providers and ED personnel play an essential role in mitigating overcrowding.

EMS providers transport patients to nearby hospitals with available beds at the expected required level of care, if necessary by diversion. In the Netherlands, diversions to redirect patients requiring care at an ED, Intensive Care Unit (ICU), coronary care unit (CCU) or general ward admission are similar to those stated in literature. [5–7] A prerequisite for an effective diversion is the capability of EMS providers to accurately predict the disposition of the patient. However, current literature regarding this topic is dated and scarce. [8–12] In these studies, patients requiring the most urgent care were excluded, which may have influenced outcome given the substantial number of excluded patients.

ED nurses and physicians can further reduce overcrowding by reducing the length of stay at the ED, and by early and accurate recognition of the patient’s disposition shortly after ED arrival. The Early Warning Score (EWS) was developed to identify clinically deteriorating patients based on their vital parameters. [13] In the Netherlands, the Modified Early Warning Score (MEWS) is the most frequently used variant of EWS. The accuracy of an EWS in general, and MEWS specifically, has never been compared to the accuracy of care providers in predicting hospital admission. However, this model is increasingly being used as a tool to predict the need for hospital admission. [14, 15] The main goal of this study was to examine and compare the accuracy of predicting the requirement for hospital admission by EMS personnel, ED nurses and physicians, and by MEWS. We hypothesized that healthcare providers have a 10% higher sensitivity and specificity compared to MEWS in predicting the need for hospital admission.

## Methods

### Study design

A prospective, observational study was performed at Amsterdam-UMC location AMC and VUmc. Both hospitals serve as Level 1 trauma centres, each providing emergency care for approximately 25,000 patients per year. Data were collected from the 11th of March to the 29th of October 2021. The study protocol has been published prior to start of the study, https://www.onderzoekmetmensen.nl/nl/trial/26360.

### Ethics approval and consent to participate

The medical ethics committee of AmsterdamUMC waived the ethical approval of the study (waiver: W-19_480 # 19.554) and consent to participate was therefore not necessary.

### Endpoints

The primary endpoint of this study was the accuracy of the prediction of hospitalization (admission versus discharge) by the EMS personnel, ED nurses and physicians compared to the accuracy of prehospital MEWS. As secondary endpoints the effects of post-graduate working experience on the primary outcome and the accuracy of predicting the need for ICU admission within 72 h were assessed.

### Patient selection

All EMS personnel was eligible for inclusion when transporting an adult patient (18 years or older) to the ED during data collection sessions. All ED nurses and physicians present at the briefing by the EMS were eligible for inclusion. All patients were included regardless of the nature of the presentation (medical or trauma). Interhospital transferred patients and patients receiving prehospital cardiopulmonary resuscitation (CPR) were excluded.

### Sample size calculation

Our primary hypothesis is that healthcare providers have a significantly higher specificity and sensitivity (of 10%) compared to MEWS > 3 in predicting the need for hospital admission. Based on this assumption with a 95% confidence interval and 80% power and an effect size of 0.2, 199 observations were needed to test our hypothesis.

### Data collection and analysis

Patients were included during workhours between 10 AM and 6 PM, which corresponds with peak hours of ambulance arrival. [4] Upon arrival of a patient by ambulance, one of three researchers noted the type of presentation, the demographics of the patient and prehospital vital parameters on a standardized form. Type of presentation included the location of care (e.g., trauma bay, coronary care unit, regular ED room); if prehospital notice was sent to the ED; and prehospital involvement of a physician such as a general practitioner.

Using a structured interview, all involved healthcare providers were asked to predict the patient’s disposition (discharge, admission). All healthcare providers were interviewed as soon as possible after the briefing, without hindering patient care.

Actual dispositions and resuscitation policies were assessed 24 h after ED presentation, using the electronic patient data management system (EPIC systems corp., Verona, Wisconsin, USA). 72 h after presentation the disposition was verified again to determine any indirect ICU admissions.

### Data and statistical analysis

All data were entered in a predefined data collection system (Castor edc). Data and statistical analyses were performed using SPSS Statistics for Windows, version 26.0 (IBM corp., Armonk, New York, USA). Data is available upon reasonable request, please contact l.i.veldhuis@amsterdamumc.nl.

Prehospital MEWS was calculated according to the collected vital parameters from ambulance handover. The components of MEWS and its scoring system used are shown in Table 1. *Of note, not all parameters (including ‘feeling worried’ or urine production are measured and therefore the MEWS that is calculated can potentially be lower in relation to the ‘true’ patient condition).*

**Table 1:** MEWS score

	3	2	1	0	1	2	3
Breathing frequency (per minute)		< 9		10–14	15–20	21–30	> 30
Oxygen saturation (with therapy, %)	< 90						
Heart rate (bpm)		< 40	40–50	51–100	101–110	111–130	> 130
Systolic blood pressure (mmHg)	< 70	70–80	81–100	101–200		> 200	
Temperature (ºC)		< 35,1	35,1–36,5	36,6–37,5	> 37,5		
Consciousness (AVPU)				A	V	P	U

*Abbreviations*: *AVPU = Alert-Verbal-Pain-Unresponsive score, bpm = beats per minute*

For continuous and not normally distributed data median and interquartile ranges (IQR) were presented. For nominal and ordinal data, absolute values and percentages were presented. Mann-Whitney U test was used to determine the difference between ordinal, and continuous, not normal distributed data. Chi-square of homogeneity and Fisher’s exact test were used to determine the difference between nominal data.

Sensitivity, specificity, positive predictive value (PPV) and negative predictive value (NPV) with 95% confidence intervals (CI) were calculated for both the prediction of hospital admission in general and of ICU-admission within 72 h of ED presentation.

Inter-observer agreement between healthcare providers was tested using Fleiss’ kappa statistics. Cut-off points for Fleiss’ kappa are: < 0.20 poor, 0.21–0.40 fair, 0.41–0.60 moderate, 0.61–0.80 good and 0.81-1.00 very good. [16] Logistic regression was used to determine the influence of years of working experience on the accuracy of the prediction (discharge versus admission). For all tests, an alpha of 0.05 was considered statistically significant.

### Bias reduction

Bias was reduced as much as possible. Selection bias was reduced by including ambulances based on time of arrival, regardless of the severity of illness or other characteristics. Healthcare providers were interviewed without the presence of others, to prevent external influence. Also, to reduce information bias, the interviews of ED nurses and physicians took place after the quick initial assessment of the patient. Only prehospital vital signs were included as parameters, to ensure equality in information availability among the healthcare providers interviewed.

## Results

In total, medical personnel were interviewed regarding 798 patients that were all included for analysis. Of these 798 patients, 405 patients were discharged (50.8%) and 393 patients were admitted to the hospital (49.2%). 334 patients were admitted to a general ward (41.9%) and 59 to the ICU within 72 h (7.4%). Patient characteristics are displayed in Table 2. The interviewed healthcare providers were predominantly EMS personnel (*n* = 796) and ED nurses (*n* = 741). The physicians’ group (*n* = 205) consisted of 106 ED physicians (51,7%) and 99 residents (48.3%).

**Table 2:** Patient characteristics of discharge versus admission

	ED discharge		Hospital admission		Discharge versus admission	
Patients, n (%)	405	(50.8)	393	(49.2)		
Age, median (IQR)	65	(49–77)	68	(55.5–77)	*p* = 0.04	
Male, n (%)	217	(53.6)	226	(57.5)	*p* = 0.27	
Place of residence, n (%)						
- Independent - Nursing home - Physical rehabilitation center	366 28 11	(90.4) (6.9) (2.7)	361 22 10	(91.9) (5.6) (2.5)	*p* = 0.53 *p* = 0.47 *p* = 1.00	
Prehospital alert, n (%)	259	(64.0)	313	(79.6)	*p* < 0.001	
Primary care physician involved prehospital, n (%)	189	(46.7)	201	(51.1)	*p* = 0.21	
Prehospital MEWS, median (IQR)	1	(0–2)	2	(1–4)	*p* < 0.001	
Location of resuscitation						
- Regular ED room - Resuscitation bay - Trauma bay - Acute brain care unit - Coronary Care unit	237 12 35 41 80	(58.5) (3.0) (8.6) (10.1) (19.8)	219 19 57 68 30	(55.7) (4.8) (14.5) (17.3) (7.6)		
Medical personnel present initially and interviewed, n (%)						
- EMS provider - ED nurse - Physician	404 376 94	(99.8) (92.8) (23.2)	392 365 111	(99.7) (92.9) (28.2)	*p* = 0.74 *p* = 0.98 *p* = 0.10	
28-day mortality, n (%)	8	(2.0)	49	(12.5)	*p* < 0.001	

*Abbreviations*: *ED =, Emergency Department, EMS = Emergency Medical Services, IQR = interquartile range, MEWS = modified early warning score*

### Primary outcome: predicting hospital admission (discharge versus admission)

EMS providers, ED nurses and physicians had a sensitivity of 82.4%, 80.0% and 91.9% in predicting hospital admission, and specificity was 56.4%, 63.0% and 67.0% respectively. MEWS ≥ 3 had a sensitivity of 44.0% and specificity of 75.6%. The sensitivity was significantly better for all healthcare providers compared to MEWS ≥3 (*p* < 0.001). However, specificity was significantly lower compared to MEWS ≥ 3 (*p* < 0.05). Table 3 shows the sensitivity, specificity, PPV and NPV of the prediction of hospital admission by healthcare providers and MEWS ≥3.

**Table 3:** Predicting hospital admission (hospital admission versus discharge)

	EMS provider (*n* = 796)			ED nurse (*n* = 741)		Physician (*n* = 205)		MEWS ≥ 3 (*n* = 651)	
Sensitivity (95% CI)	82.4%	(78.3–86.0)	80.0%		(75.5–84.0)	91.9%	(85.2–96.2)	44.0%	(38.6–49.6)
Specificity (95% CI)	56.4%	(51.4–61.3)	63.0%		(57.9–67.9)	67.0%	(56.6–76.4)	75.6%	(70.6–80.2)
PPV (95% CI)	64.7%	(61.9–67.4)	67.7%		(64.5–70.7)	73.0%	(66.8–78.4)	63.6%	(58.2–68.7)
NPV (95% CI)	76.8%	(72.4–80.7)	76.5%		(72.3–80.2)	89.5%	(81.8–94.2)	58.3%	(55.5–61.0)

*Abbreviations*: *ED = Emergency Department, EMS = Emergency Medical Services, NPV = Negative Predictive Value, PPV = Positive Predictive Value, 95% CI = 95% confidence intervals*

Hospital admission was associated with more frequent prehospital notification of arrival by EMS, higher prehospital MEWS, and increased 28-day mortality (all *p* < 0.001). The performance of EMS and ED nurses was similar in predicting the need for hospital admission (*p* = 0.25). EMS, ED nurses and physicians showed similar accuracy in predicting the need for hospital discharge (*p* = 0.21). However, the sensitivity of physicians in predicting the need for hospital admission was significantly better compared to the accuracy of EMS and ED nurses, (*p* < 0.001). Fleiss’ kappa for inter-observer agreement for hospital admission was 0.40 (*p* < 0.001) and discharge 0.34 (*p* < 0.001). Both Kappa’s are classified as fair agreement.

### Secondary outcome: predicting ICU admission

Actual ICU admission rate within 72 h was 59 out of 393 patients (15.0%). Table 4 shows the accuracy for predicting need for ICU admission within 72 h by healthcare providers and MEWS ≥ 3. No significant differences were found in predicting the need for ICU admission by healthcare providers and/or MEWS ≥ 3. All groups of healthcare providers were significantly better at predicting not needing ICU admission (thus needing ward admission) compared to MEWS (all *p* < 0.001).

**Table 4:** Accuracy at predicting the need for ICU admission within 72 h (ICU versus ward admission)

	EMS provider (*n* = 325)		ED nurse (*n* = 292)		Physician (*n* = 102)		MEWS ≥ 3 (*n* = 327)	
Sensitivity (95% CI)	57.6%	(44.1–70.4)	64.7%	(50.1–77.6)	48.3%	(29.5–67.5)	73.3%	(58.1–85.4)
Specificity (95% CI)	94.7%	(91.3–97.1)	97.1%	(94.1–98.8)	95.9%	(88.5–99.1)	60.6%	(54.7–66.4)
PPV (95% CI)	46.7%	(33.4–60.4)	64.0%	(45.5–79.2)	48.4%	(22.6–75.2)	13.0%	(10.6–15.8)
NPV (95% CI)	96.6%	(95.4–97.7)	97.2%	(96.0–98.0)	95.9%	(94.2–96.7)	96.6%	(94.6–97.9)

*Abbreviations*: *ED = Emergency Department, EMS = Emergency Medical Services, NPV = Negative Predictive Value, PPV = Positive Predictive Value, 95% CI = 95% confidence interval*

### Secondary outcome: correlation with work experience

Median years of working experience were 10 years for EMS providers (IQR 4–20), 5 years for ED nurses (IQR 3–12) and 5 years for physicians (IQR 3–11). Using logistic regression, the number of years of working experience had a significant correlation with correctly predicting the need for hospital admission or discharge in the group of physicians (*p* < 0.001), not in the group of EMS (*p* = 0.32) and ED nurses (*p* = 0.90).

## Discussion

### Summary and comparison with existing literature

In this multicentre, observational study, it was shown that healthcare providers can accurately predict the need for hospital admission with a sensitivity of 80–92% and specificity of 56–67%. Although patients requiring hospital admission had a significantly higher MEWS score, the predictive capabilities of healthcare providers regarding hospital admission greatly exceeded the performance of MEWS ≥ 3 (80–92% versus 44%;). Hereby healthcare providers have a sensitivity higher than hypothesized, and a lower specificity than expected, as well as a significantly better sensitivity compared to MEWS ≥ 3. When interpreting the results found in this study, the sensitivity of the predictions is deemed most important in order not to miss any patient requiring hospitalization, i.e., to minimize undertriaging. Focusing on the specificity, i.e., accurately recognizing patients who do not require hospitalization, would lead to a different approach in solving ED overcrowding, but would not result in better outcomes related to health and mortality.

The sensitivity of the EMS and ED nurses’ predictions found (82% and 80% respectively) were better when compared to existing literature. [8–12, 17, 18] These findings might suggest that the high training standards of both the EMS and ED nurses in the Netherlands resulted in more accurate predictions [19, 20] The physicians showed the lowest percentages of undertriaging regarding hospital admission (8%), performing significantly better than EMS and ED nurses. Comparing the sensitivity of the physicians’ predictions to current literature, these findings are also higher than expected. [18, 21, 22] This is despite both studies having a similar number of interviews and both interviewing residents as well as medical specialists. [18, 21, 22] No consensus exists regarding acceptable rates for under- and overtriaging, complicating the interpretation of the findings of this study. However, similar percentages to the results of the EMS have been accepted as reasonable and the rate of undertriaging by physicians is considered acceptable without controversy. Thus, these findings suggest that Dutch healthcare providers performed above average in accurately predicting the need for hospital admission. [8, 10–12, 23]

As for the differences in attendance between healthcare providers, physicians were predominantly present at the EMS briefing in case of a 112-notification (European emergency number and ambulance service, like 911). Consequently, physicians were interviewed considering a different and potentially more severely ill population compared to EMS personnel and ED nurses. The latter also attended patients presented based on referral by general practitioners. This might partially explain the higher overall accuracy on the predictions made by physicians compared to EMS personnel and ED nurses (Table 3). However, even when EMS personnel, ED nurses and physicians are compared amongst each other based on the same patient population, this difference persists. This might be explained by differences in training and emphasis of their respective education.

Although unable to accurately predict hospital admission in general, MEWS has shown to moderately improve accuracy regarding the prediction of which patient requires ICU admission. The ability of MEWS to identify patients requiring ICU admission within 72 h found is considered reasonable, with a sensitivity of 73% and specificity of 61%. Important to note is that the MEWS value calculated in this study may underappreciate the true condition of the patient as some parameters including urine production or ‘feeling worried’ have not been documented as they are not part of regular assessment by EMS personnel. This study is the first to compare healthcare providers to the MEWS score in predicting the need for ICU admission. The cut-off value used was chosen according to the standard hospital protocol of the centres included in this study. Despite the cut-off value used being lower than described in other literature regarding MEWS performance in general, the accuracy found is comparable and is thus deemed representative. [24, 25]

All healthcare providers performed excellently at predicting the need for ward admission specifically, resulting in a significantly lower percentage of undertriaging than MEWS (undertriaging 3–5% versus 39%). These results suggest that the assessment of EMS personnel, ED nurses and physicians is superior to that of MEWS in identifying patients requiring ward admission. However, it should be noted that this study is not powered based on this secondary endpoint and thus further sufficiently powered research on this topic is needed.

### Strengths and limitations

This multicentre study is the first to compare the accuracy of predictions made by both prehospital and ED healthcare providers. Also, the comparison between the three groups of healthcare providers and MEWS scores in accurately predicting the need for ICU admission has not been made before. A sound methodology was used, including repeated checks of the data collected, resulting in a very low drop-out rate. Several limitations of this study should be mentioned. Selection bias cannot be ruled out due to the timing of data collection sessions. These sessions aimed at including as many patients as possible during peak hours of ambulance arrival. [4] To what extent this timeframe may have had on the outcome parameters is unknown. We can hypothesize that patients presented at evening or nighttime maybe sicker but may also have more logistical/social problems requiring a hospital admission not for a strict medical reason perse.

Given the observational nature of this study, an effort was made not to hinder (urgent) patient care. Regarding the small difference in accuracy between EMS personnel and ED nurses, it should be considered that most of the Dutch EMS personnel has worked as an ED nurse in the past, and a minority currently still does. This interferes with the comparison that can be made between these two groups.

In this study, patients with restricted treatment policies during their admission were still included. 57 of the admitted patients (15%) had an extensively restricted treatment policy, consisting of do not resuscitate and no ICU admission. Two patients with no ICU admission policy died within three days. Thus, it cannot be ruled out that these patients needed ICU admission/support. Since this entails only two patients, the effect of these restricted treatment policies on the accuracy of predicting the need for ICU admission is deemed small. Also, as hospitals wards are increasingly getting busier, admission to a hospital ward maybe delayed. Since we checked admission after 72 h of ED presentation, this potential outcome bias is not present.

As this study only included patients arriving by ambulance and are likely to have a higher severity of illness, these results can’t be translated to all (other) ED patients. The majority of patients come to ED by their own means and are either send by their health care provider or come at their own discretion.

## Conclusions

This prospective multicentre study has demonstrated that EMS personnel, ED nurses and physicians can accurately predict which patients require hospital admission directly after EMS briefing at the ED. All healthcare providers outperformed the MEWS on predicting hospital admission. In addition, the EMS, ED nurses and physicians can excellently predict which patients will not require ICU admission within 72 h. The underlying reasons for the differences observed between the types of health care workers are not understood at this time and warrant future investigations.

*This has great relevance for efficient triage and throughput at the ED as the clinical judgement of primarily the ED physician is very important at time of stress and high occupancy.* Therefore, hospital admission can be initiated as soon as the patient enters the ED if the physician predicts that admission will be necessary.

### Electronic supplementary material

Below is the link to the electronic supplementary material.

Supplementary Material 1

## Data Availability

Data is available upon reasonable request.

## Abbreviations

AVPU score: alert, verbal, pain, unresponsive score
BCU: brain care unit
CCU: coronary care unit
CI: confidence interval
ED: emergency department
EMS: emergency medical services
EWS: early warning score
ICU: intensive care unit
MEWS: modified early warning score
NPV: negative predictive value
PPV: positive predictive value
