# Supplementary material for: The accuracy of predicting hospital admission by emergency medical service and emergency department personnel compared to the prehospital MEWS: a prospective multicenter study
Source: BMC Emerg Med. 2024 Jul 9;24:111. doi: 10.1186/s12873-024-01031-9 (PMC11234550; doi:10.1186/s12873-024-01031-9)
Supplement: Supplementary file 1 — Supplementary Material 1 [file 12873_2024_1031_MOESM1_ESM.docx]

Supplementary file 1. Inclusion document

| **Inclusion number** |  |
| --- | --- |
| **Day of presentation** | ____/____/____ |
| **Time of presentation** | ____:____ |

| **Vital parameters** | **EMS:** |
| --- | --- |
| Respiratory rate |  |
| Saturation without oxygen |  |
| Saturation with oxygen |  |
| Blood pressure |  |
| Pulse rate |  |
| Temperature |  |
| Glasgow coma scale |  |

| **Judgement** | **Home / admission or intensive care** |
| --- | --- |
| Ambulance provider |  |
| ED nurse |  |
| ED physician |  |

**Day one**

| **Admission** | Yes / No |
| --- | --- |
| **Admission in case yes** | Ward / intensive care |
